# *Salix daphnoides*: A Screening for Oligomeric and Polymeric Proanthocyanidins

**DOI:** 10.3390/molecules200813764

**Published:** 2015-07-29

**Authors:** Stefan Wiesneth, Frank Petereit, Guido Jürgenliemk

**Affiliations:** 1 Institute of Pharmaceutical Biology, Universitätsstr. 31, Regensburg D-93053, Germany; E-Mail: guido.juergenliemk@chemie.uni-regensburg.de; 2 Institute of Pharmaceutical Biology and Phytochemistry (IPBP), Corrensstr. 48, Münster D-48149, Germany; E-Mail: frank.petereit@uni-muenster.de

**Keywords:** procyanidins, proanthocyanidins, flavan-3-ols, *Salix daphnoides*, willow bark, low temperature NMR, thiolysis, polarimetry, polymeric proanthocyanidins

## Abstract

In the present study, a qualitative analysis of proanthocyanidins (PAs) from an aqueous-methanolic extract of *Salix daphnoides* VILL. bark is described. Procyanidin B1 (**1**), B2 (**2**), B3 (**3**), B4 (**4**), C1 (**5**), epicatechin-(4*β*→8)-epicatechin-(4*β*→8)-catechin (**6**) and epicatechin-(4*β*→8)-epicatechin-(4*β*→8)-epicatechin-(4*β*→8)-catechin (**7**) have been isolated by a combination of different chromatographic separations on Sephadex^®^ LH-20-, MCI^®^-, Diol-and RP-18-phases. Mass spectrometry, 1D- and 2D-NMR, circular dichroism and polarimetry were used for their structure elucidation and verification by comparison with the literature. Additionally, two fractions of very polar flavan-3-ols were compared: “regular” polymeric PAs received at the very end of the Sephadex^®^ LH-20 chromatography showing no mobility on silica TLC and “unusual” PAs with the same R_F_-value but already eluting together with flavonoids in the Sephadex^®^ LH-20 system. These “unusual” PAs were subsequently enriched by centrifugal partition chromatography (CPC). ^13^C-NMR, polarimetry, thiolysis, acid hydrolysis and phloroglucinol degradation were used to characterize both fractions. Differences in the composition of different flavan-3-ol units and the middle chain length were observed.

## 1. Introduction

Willow bark (*Salicis cortex*, *Salix* spp., Salicaceae) is an herbal drug monographed in the European Pharmacopoeia (Ph. Eur.) [1]. It is used for its antiphlogistic, antipyretic and analgetic effects and is positively monographed by the European Scientific Cooperative On Phytotherapy (ESCOP) for the treatment of rheumatic diseases, fever and headache [2,3]. Since the monograph of willow bark in the Ph. Eur. [1] does not claim a specific willow species for the drug, but insists on a minimum amount of 1.5% salicylic alcohol derivatives, there is a great variety in willows which can be used for the drug Salicis cortex. Although it is known that these salicylic alcohol derivatives are metabolized to the anti-inflammatory compound salicylic acid *in vivo* [4,5], there is doubt that only this class of secondary plant ingredients is responsible for the effects described above [2,3] as the resulting *in vivo* concentration is much too low to explain the overall efficacy of willow bark [5]. As not only anti-inflammatory but also cooperative effects are described for proanthocyanidins (PA) [6,7], which are one of the main groups of *Salix* ingredients beside the salicylic alcohol derivatives and flavonoids [3], it is necessary to investigate each willow species to broaden the knowledge about the complete phenolic spectrum of these important compounds.

The aim of the present study was to investigate the proanthocyanidin composition of *Salix daphnoides* VILL., the European violet willow, which is one of the main *Salix* species used for the drug willow bark due to the European Pharmacopoeia [1]. Pobłocka-Olech and coworkers could identify procyanidin B1 by SPE-HPTLC in *S. daphnoides* [8], but a systematic investigation of the complete proanthocyanidin pattern is still missing.

## 2. Results and Discussion

### 2.1. Screening for Oligomeric Proanthocyanidins

**Figure 1:**
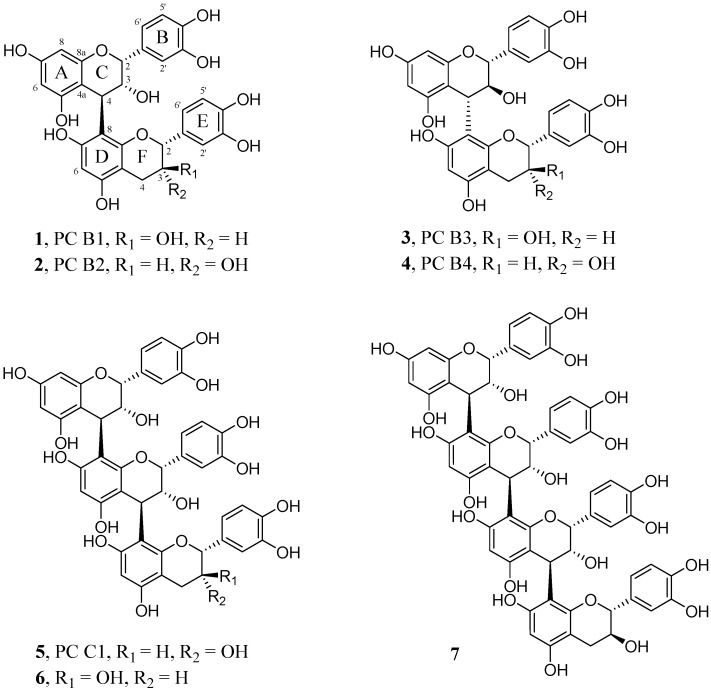
Structures of the isolated procyanidins (PC) **1**–**7**.

To broaden the knowledge about the composition of oligomeric flavan-3-ols, 100.00 g of a *S. daphnoides* bark extract were used for their isolation. Compounds **1**–**4** eluted from Sephadex^®^ LH-20 as stationary phase between 2200 and 2986 mL (fraction S 3.2), compounds **5** and **6** between 2986 and 4154 mL (fraction S3.3), and compound **7** between 4154 and 4634 mL (fraction S3.4) (Figure 1). Each of these fractions were further separated by MCI^®^-Gel chromatography and finally purified by preparative HPLC (system A: **1**, **3**; system B: **2**, **4**; system C: **5**, **6**; system D: **7**).

The dimeric procyanidins B1 (**1**), B2 (**2**), B3 (**3**) and B4 (**4**) were confirmed by comparing the spectroscopic data with results obtained by Shoji *et al.* [9]: B1 and B2, Kolodziej [10] B1, peracetylated, Saito *et al.* [11]: B3, [12]: B4, and Mohri *et al.* [13]: B3 and B4. CD spectroscopy was used to verify the 4*α*→8 or 4*β*→8 linkage between the flavan-3-ol units. High-amplitude positive Cotton effects at low wavelengths (220–240 nm) indicate a 4*β* and negative effects a 4*α* configuration [14,15,16,17]. The CD-spectra for the isolated compounds were in accordance with the results published by Barrett *et al*. [15]: B2, B3 and B4. Since there were no CD data available for **1** and **7** as free phenolic compounds in literature (SciFinder^®^, American Chemical Society, Washington, DC, USA), this general rule was applied to determine the configuration of C-4 in their upper units (Supplementary Figures S1 and S2). As observed by Barrett and coworkers [15], mol. Ellipticity [Θ] of the respective maximum with positive sign rises with an increasing number of monomeric units for these compounds. Thus, the absolute stereochemistry at the interflavan linkages was assigned as 4*β*→8 in **7**.

NMR data sets (^1^H, ^13^C) of **3** and **4** (free phenolic) are given in Supplementary Table S1. The ^1^H-NMR data obtained for compound **5** (procyanidin C1, epicatechin-(4*β*→8)-epicatechin-(4*β*→8)-epicatechin) at −20 °C were in accordance with Shoji *et al.* [9], except the coupling constant for H-2′ at δ 7.02 ppm for the middle unit. Whereas Shoji and coworkers found a doublet with *J* = 8 Hz [9], a typical *meta* coupling constant of *J* = 1.6 Hz was found in the present work. The optical rotation and CD-data were in accordance with the literature [15,18,19]. To complete the NMR data, spectra at −40 °C were determined (Supplementary Table S2). The ^1^H-NMR data of compound **6** (epicatechin-(4*β*→8)-epicatechin-(4*β*→8)-catechin) measured at −20 °C are very similar to a trimer identified by Shoji *et al.* [9]. Whereas Shoji *et al*., published δ 4.06 ppm (d, *J* = 8 Hz) for H-3 of ring F and δ 3.99 ppm (d, *J* = 5 Hz) for the respective C ring proton, δ 4.07 ppm (d, *J* = 1.3 Hz) and δ 3.99 ppm (d, *J* = 1.8 Hz) were found in the present study for the same protons. The small coupling constants of both H-3 protons indicate the 2,3*-cis* relative configuration and thus epicatechin as constituent units. CD data were in agreement with published values [19]. NMR data at −40 °C are given in Supplementary Table S2. Compound **7** was determined as epicatechin-(4*β*→8)-epicatechin-(4*β*→8)-epicatechin-(4*β*→8)-catechin by comparing the ^1^H-NMR data measured at −20 °C with the data of Saito *et al.* [20]. The ^1^H-NMR data agree with published data except a 0.08 ppm lower shift for H-2 of the terminal flavan-3-ol unit (δ 5.04 ppm). NMR data sets at −40 °C and −20 °C are presented in Supplementary Table S3.

### 2.2. Characterization and Comparison of Fractions Enriched with Oligomeric or Polymeric Proanthocyanidins

Sephadex^®^ LH-20 was used as the first stationary phase for column chromatography to separate an extract of *S. daphnoides*. Using 70% ethanol as mobile phase, the salicylic alcohol derivatives were eluted first (S1), followed by the flavonoids (S2) and the oligomeric proanthocyanidins (PAs) in the last fraction (S3). Polymeric PAs eluted after changing the mobile phase to 70% acetone (S3) [21]. Using vanillin/HCl-spray reagent for the detection of PAs in the TLC control, not only very polar and thus immobile red colored “regular” polymeric PAs were observed at the end of the Sephadex^®^ chromatography with 70% acetone but also similar polar “unusual” proanthocyanidins with an identical chromatographic behavior in the same TLC-system (R_F_ = 0) were eluted already with 70% ethanol together with flavonoids, even before the first monomeric flavan-3-ol. Those Sephadex-fractions were combined and CPC (centrifugal partition chromatography) with a 1-butanol/water system was used to remove most of the flavonoids to enrich the “unusual” PAs in the water phase (S2 W) in order to compare them with the “regular” PAs (S3.8).

Parameters for the comparison of the “unusual” and “regular” PAs were the content of tannins [22,23], the content of procyanidins (PCs) [24] and the determination of the corresponding anthocyanidins yielded by the oxidative cleavage reaction [25]. Further, the mean degree of polymerization (mDP) was estimated by thiolyses [26,27] and spectroscopic data obtained by ^13^C-NMR [28,29,30], circular dichroism (CD) and polarimetry [21,31] were compared.

The total content of tannins of both fractions was determined by a modified Folin-Ciocalteu reagent assay of the Ph. Eur. [22]. With this functional assay it is possible to determine the content of both, hydrolyzable and condensed tannins. Since it is a convention method, the values generated may not represent the “real” content of tannins, but it is very suitable for a comparison. To get more information about the adsorption properties of the fractions, tannic acid was used as a 100% reference. The fraction with the “unusual” PAs accomplished a content of 66.5% tannins and the “regular” PAs 84.0%. In the monograph “Crataegi fructus” of the Ph. Eur. [24], another convention method is described to determine the content of procyanidins (PCs). An oxidative cleavage reaction with HCl allows the calculation of the procyanidin content as cyanidin chloride by photometry. Using this method, 57.9% PCs were determined in the fraction with the “unusual” PAs and 78.2% with the “regular” PAs. Thus, both methods revealed a slightly higher content of tannins in the fraction with the “regular” PAs.

After the oxidative cleavage reaction, the corresponding anthocyanidins were determined by HPLC using a method of Zhang and coworkers [25] with pelargonidin chloride, cyanidin chloride and delphinidin chloride as reference compounds. To estimate their concentration, the corresponding areas were integrated at 525 nm and set as 100%. Main compound of both fractions was cyanidin (94.3% and 97.8%, respectively) indicating that proanthocyanidins with dihydroxylated B-ring flavan-3-ol constituent units like catechin and epicatechin are dominating. Only small amounts of pelargonidin and delphinidin were identified as cleavage products but the content of delphinidin was slightly higher in the fraction with the “regular” PAs (4.3%) than in the fraction with the “unusual” PAs (0.8%, Table 1) indicating more B-ring trihydroxylated flavan-3-ol constituent units in this fraction. HPLC-chromatograms are shown in Supplementary Figure S3. These results were also confirmed qualitatively by LC-ESI-HRMS.

**Table 1:** Estimation of mono-, di-, and trihydroxylated anthocyanidins.

Fraction	Delphinidin (R_T_ = 5.91 min)	Cyanidin (R_T_ = 10.07 min)	Pelargonidin (R_T_ = 18.28 min)
“regular” PAs	4.3%	94.3%	1.4%
“unusual” PAs	0.8%	97.8%	1.4%

A further estimation of the hydroxylation pattern and the mean degree of polymerization (mDP) of both fractions was determined by thiolysis with benzyl mercaptan and subsequent HPLC analysis [26,27] using the method of Dauer and coworkers [26]. The identification of reaction products was confirmed by LC-ESI-HRMS. The cleaved terminal units are the unsubstituted flavan-3-ols catechin and epicatechin (peaks 1 and 2). The upper units were identified as thioethers (R_T_ at 36.9 to 46.1 min), corresponding to flavan-3-ols and dimeric PAs substituted with benzyl mercaptan in position C-4. Table 2 gives an overview of the detected products yielded from the thiolysis (chromatograms are shown in Supplementary Figure S4). The main reaction products of both fractions are thioethers of dihydroxylated flavan-3-ols (peaks 3 **, 5, 6, 7 **, 8 * and 9). Only in the fraction with the “regular” PAs, peak 4 * could be found which corresponds to a thioether of a trihydroxylated flavan-3-ol (*m*/*z* 427.0862). Peak 10 (*m*/*z* 395.0963) indicates the presence of flavan-3-ols with monohydroxylated B-rings in both fractions. These results are in accordance with the findings of the determination of anthocyanidins after cleavage reaction with HCl/O_2_. The difference in the mDP of both fractions is obvious: whereas the mDP in the fraction with the “unusual” PAs was 4.9, a value of 11.8 could be determined for the fraction with the “regular” PAs.

**Table 2:** Thiolysis related compounds.

Peak	R_T_ (min) (LC-HRMS)	R_T_ (min) Anal. HPLC	*m*/*z* [M − H]^−^	Chemical Formula (calcd [M − H]^−^)
1	20.97	22.82	289.0725	C_15_H_14_O_6_ (289.0718)
2	23.25	25.14	289.0723	C_15_H_14_O_6_ (289.0718)
3 **	35.06	36.92	699.1544	C_37_H_32_O_12_S (699.1542)
4 *	36.60	38.40	427.0862	C_22_H_20_O_7_S (427.0857)
5	37.24	39.08	699.1548	C_37_H_32_O_12_S (699.1542)
6	39.08	40.81	699.1545	C_37_H_32_O_12_S (699.1542)
7 **	39.33	41.71	699.1540	C_37_H_32_O_12_S (699.1542)
8 *	40.50	42.19	699.1543	C_37_H_32_O_12_S (699.1542)
9	41.39	43.12	411.0908	C_22_H_20_O_6_S (411.0908)
10	44.45	46.12	395.0963	C_22_H_20_O_5_S (395.0959)

* Only found in the chromatogram of the “regular” PAs; ** Only found in the chromatogram of “unusual” PAs.

**Figure 2:**
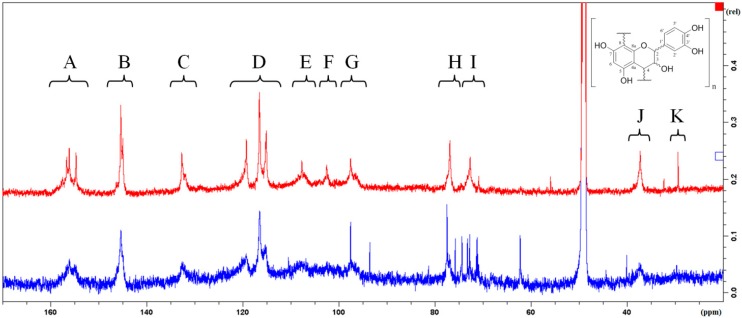
^13^C-NMR of the “regular” (upper) and the “unusual” (lower) PAs. A (152–160 ppm): C-5, C-7, C-8a; B (144–147 ppm): C-3′, C-4′; C (131–134 ppm): C-1′; D (114–123 ppm): C-2′, C-5′, C-6′; E (106–110 ppm): substituted C-6 and C-8; F (101–104 ppm): C-4a; G (95–99 ppm): unsubstituted C-6 and C-8; H (76–78 ppm): C-2; I (71–74 ppm): C-3; J (36–39 ppm): C-4 of extender units; K (29–30 ppm): C-4 of terminal unit.

^13^C-NMR is an established method for the characterization of PA fractions [21,28,29,30]. Figure 2 procures an overview of the ^13^C-NMR spectra of the “regular” and “unusual” PAs. The ratio of the integrated signals K(C-4 of terminal units) and J (C-4 of extender units) of the “regular” PAs is 1:11 indicating a middle chain length of 12 constituent flavan-3-ol units which is in accordance to the results obtained for the determination of the mDP by thiolysis. However, this result is in contrast to former results obtained with “regular” PAs of *Salix purpurea* L. with the same method as a mDP of 4–5 flavan-3-ol units could be calculated [30]. Unfortunately, the signal of C-4 of the terminal unit in the ^13^C-spectrum of the “unusual” PAs is too weak for an exact integration.

The signals between 62.0 and 76.0 ppm in the spectrum of the “unusual” PAs (Figure 2, lower spectrum) seem to be typical sugar signals. But as no flavan-3-ol-glycosides could be found in any HPLC-MS experiment after a cleavage reaction (thiolysis, HCl/O_2_ and phloroglucinol degradation), they should be due to very polar impurities of S2 W which could not be removed by CPC.

**Figure 3:**
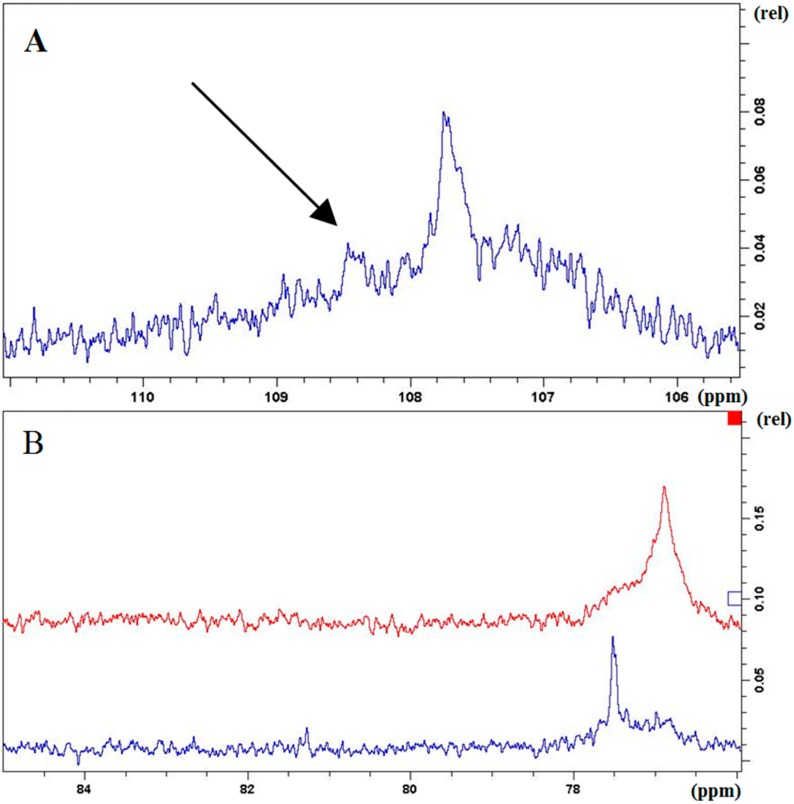
**A**: Signal at 108.4 ppm in the ^13^C-spectrum of the “regular” PAs indicating the presence of monomeric flavan-3-ols with trihydroxylated B-rings; **B**: 76–85 ppm of the ^13^C-NMR spectra of the “regular” (**upper**) and “unusual” (**lower**) PAs. The area of 76–78 ppm is representative for the C-2 of 2,3*-cis* configurated flavan-3-ol units. The signals of 2,3*-trans* configurated units would appear in between 82 and 84 ppm.

In the spectrum of the “regular” PAs, a small signal at 108.4 ppm could be observed corresponding to C-2ʹ and C-6ʹ of trihydroxylated flavan-3-ols (Figure 3A). This is again in accordance to the former results obtained by the cleavage reactions by HCl/O_2_ and benzyl mercaptan.

Furthermore ^13^C-NMR enables the possibility to get structural information about the relative configuration of the constituent flavan-3-ol units. The signal cluster from 76 to 78 ppm (Figure 3B) can be assigned to the C-2 carbons of 2,3*-cis* configurated flavan-3-ols. The absence of signals from 82 to 84 ppm (signals for 2,3*-trans* flavan-3-ols) indicates that units with 2,3*-cis* configuration are predominant in both fractions.

This was verified by optical rotation for the fraction with the “regular” PAs (89.9%) [21,31]. As the fraction containing the “unusual” PAs was too colored to get valid data by polarimetry, CD spectroscopy was used as a third tool to determine the C-ring stereochemistry. The positive Cotton effect between 220 and 240 nm indicated the predominance of *β*-configurated interflavan linkages and thus the predominance of 2,3*-cis* relative configurated flavan-3-ols [14,15,16,17].

## 3. Experimental Section

### 3.1. Reagents and Standards

Methanol, acetonitrile, formic acid, acetic acid, hydrochloric acid, Folin-Ciocalteu’s reagent, dried pyridine, phloroglucinol and acetic anhydride were obtained from Merck Chemicals GmbH (Darmstadt, Germany). Acetone, 1-butanol, benzyl mercaptan, methanol-*d*_4_, trifluoroacetic acid, pelargonidin chloride, delphinidin chloride (Fluka, Neu-Ulm, Germany), cyanidin chloride ≥90% (FLUKA), vanillin (Fluka), p-anisaldehyde, and d-(−)-salicin were purchased from Sigma-Aldrich Chemie GmbH (Taufkirchen, Germany). Tannic acid, sodium carbonate anhydrous, and catechin were acquired from Carl Roth GmbH & Co. KG (Karlsruhe, Germany). Chloroform-*d*_1_, and deuterium oxide were from Deutero GmbH (Kastellaun, Germany). Ethyl acetate was from Acros Organics (Geel, Belgium). The water for analysis was generated by an Astacus Reagent bench (MembraPure GmbH, Henningsdorf/Berlin, Germany). Naringenin-7-*O*-glucoside was obtained from Extrasynthese (Lyon, France). Freiberg hide powder was purchased from the Research Institute for Leather and Plastic Sheeting GmbH (Freiberg, Germany).

### 3.2. Plant Material

The sliced bark of *S. daphnoides* (Art. 174702) was a gift from PhytoLab GmbH & Co. KG (Vestenbergsgreuth, Germany). A voucher specimen of this material is deposited at the Institute of Pharmaceutical Biology of the University of Regensburg (Regensburg, Germany).

### 3.3. Extraction Procedure

The plant material was powdered with a UZM 1 mill (Retsch GmbH, Haan, Germany) with a sieve size of 0.50 (619.8 g) and afterwards mixed with sea sand (1 + 1). Exhaustive percolation with 80% methanol revealed 143.6 g extract after evaporating and drying by lyophylisation (drug-extract-ratio = 4.32:1).

### 3.4. Sephadex^®^ LH-20

Extract (100.0 g) was separated on Sephadex^®^ LH-20 (265 g, GE Healthcare GmbH, München, Germany; column: 95 × 3 cm, flow = 1 mL/min) with 70% ethanol in three runs. The column chromatography was controlled by TLC on silica gel 60 F_254_ (Merck Chemicals GmbH; ethyl acetate/formic acid/water 90 + 5 + 5) with vanillin/HCl and anisaldehyde/H_2_SO_4_ as detection reagents. Salicin, naringenin-7-*O*-glucoside and catechin were used as reference compounds in TLC. After elution of catechin, the mobile phase was changed to 70% acetone. A salicylic alcohol derivative enriched (S1, 0–1196 mL, 75.35 g), a flavonoid enriched (S2, 1196–2045 mL, 5.14 g), and a proanthocyanidin enriched fraction (S3, 2045 mL until no more substances were detected in TLC-control, 6.59 g) were yielded.

Finally, S3 was re-chromatographed with Sephadex^®^ LH-20 column with 70% ethanol and TLC-control with ethyl acetate/formic acid/acetic acid/water (100 + 11 + 11 + 26) and vanillin/HCl for detection to yield fractions S3.1 (689.2 mg, 0–2200 mL), S3.2 (643.2 mg, 2200–2986 mL), S3.3 (748.2 mg, 2986–4154 mL), S3.4 (237.8 mg, 4154–4634 mL), S3.5 (194.6 mg, 4634–5104 mL), S3.6 (512.5 mg, 5104–6489 mL), and S3.7 (415.8 mg, 6489–8264 mL). After S3.7 the mobile phase was changed to 70% acetone to yield S3.8 with polymeric proanthocyanidins (3259.8 mg).

### 3.5. Centrifugal Partition Chromatography (CPC)

A Spot Centrifugal Partition Chromatography machine with a 250 mL rotor (Armen Intrument, Saint-Avé, France; saturated 1-butanol/water system, flow = 5 mL/min, 1500 rpm) and a 510 HPLC pump (Waters GmbH, Eschborn, Germany) was used to enrich “unusual” proanthocyanidins from 1.0 g of S2. TLC-control was the same as in the re-chromatography of S3 (see Section 3.4.). First, the system was run in the ascending mode with the upper phase (saturated 1-butanol) as mobile phase. After 1269 mL, the system was changed to the corresponding descending mode with the lower phase (saturated water) as mobile phase and 678 mL were eluted. The combined fractions of the water-phase yielded in a fraction (S2 W) enriched with the “unusual” PAs (75.1 mg).

### 3.6. MCI-Gel^®^

The re-chromatographed fractions from the Sephadex^®^ LH-20 system were further purified by MCI-Gel^®^ CHP20P (170 g, Mitsubishi Chemical Europe GmbH, Düsseldorf, Germany; column: 600 × 25 mm (BESTA-Technik für Chromatographie GmbH, Wilhelmsfeld, Germany)) with a Spot Liquid Chromatography Flash machine (Armen Intrument; flow = 7.5 mL/min, 15.5 mL per fraction). A = 20% methanol, B = 50% methanol; gradient: 0–10 min 0% B, 10–300 min 0% → 100% B, 300–360 min 100% B. Subsequently two isocratic steps for 90 min were applied. The first one used 75% methanol the second one 100% methanol as eluent. TLC-control see Section 3.5.

### 3.7. Preparative HPLC

System A: binary Varian ProStar preparative HPLC (Varian Deutschland GmbH, Darmstadt, Germany) equipped with a diode array detector and a XDB-C18 PrepHT 21.2 × 250 mm 5-Micron column with guard column (Agilent Technologies Sales & Services GmbH & Co. KG, Waldbronn, Germany) and manual injection. A = water, B = acetonitrile, flow = 17 mL/min, gradient: 0–18 min 10% B isocratic, 18–19 min 10% → 75% B, 19–21 min 75% B isocratic; 21–23 min 75% B → 10% B, 23–28 min 10% B isocratic.

System B: binary Agilent Infinity 1260 HPLC equipped with a 1260 Agilent diode array detector, a 1260 Agilent fraction collector, a 1260 Agilent manual injector and a XDB-C18 PrepHT 21.2 × 250 mm 5-Micron column (Agilent) with guard column. Eluents as in system A, flow = 25.5 mL/min, gradient: 0–13 min 5% → 15% B,13–18 min 15% B isocratic, 18–20 min 15% → 100% B, 20–25 min 100% B isocratic, 25–27 min 100% → 5% B, 27–30 min 5% B isocratic.

System C: HPLC-system as system B, but with an Uptisphere DIOL 6 µm 250 × 21.2 Prep-LC-column with guard column (Interchim, Montluçon Cedex, France). A = acetonitrile, B = 95% methanol, flow = 15 mL/min, gradient:0–5 min 5% B isocratic, 5–25 min 5% → 30% B, 25–26 min 30% → 5% B, 26–30 min 5% B isocratic.

System D: as system C but applying a 10 mL/min flow.

### 3.8. Quantification of Tannins

To quantify the tannins in PAs rich fractions, the method 2.8.14 of the European Pharmacopeia [22] has been modified. 10.0 mg of a sample or tannic acid as reference were dissolved in 100.0 mL water. This solution contains the total phenols. 10.0 mL of this “total phenols solution” (TPS) is subjected to a flask with 100 mg Freiberg hide powder and stirred for 60 min without light. This preparation was filtered and represented the “not adsorbed phenols solution” (NAPS). Either 2.0 mL TPS or NAPS together with 1.0 mL folin-ciocalteu’s reagent were diluted with a sodium carbonate solution (53.0 g sodium carbonate anhydrous diluted with water to 500 mL) to 25.0 mL. After incubation without light for 15 min, absorbance was measured at 691 nm [23] with a Novaspec II (Pharmacia LKB, Uppsala, Sweden). All experiments were peformed three times and related to the value obtained with tannic acid as 100% reference using Equation (1) to calculate the content of tannins (A = absorbance at 691 nm; m = mass):

(1) $$\text{Content of tannins }\left(\text{\%}\right)\text{= 100 × }\frac{({{\text{A}}_{\text{TPL}}-{\text{A}}_{\text{NAPS}}\text{)}}_{\text{sample}}\times {\text{m}}_{\text{tannic}\;\text{acid}}}{({{\text{A}}_{\text{TPL}}-{\text{A}}_{\text{NAPS}}\text{)}}_{\text{tannic}\;\text{acid}}\times {\text{m}}_{\text{sample}}}$$

### 3.9. Quantification of Total Amount of Procyanidins and Qualitative Evaluation of Monomeric Constitution

To quantify the procyanidins in fractions, the acid hydrolysis described of the European Pharmacopoeia in the monograph of “Crataegi fructus” [24] has been modified. A sample (10.0 mg) was dissolved in a mixture of 16.0 mL 70% ethanol, 6.0 mL HCl (70.00 g HCl diluted with water to 100 mL) and 4.0 mL water. This preparation was heated under a reflux for 80 min. After filtration and washing, the solution was diluted to 100.0 mL. 50.0 mL were evaporated to approximately 3 mL and transferred to a separating funnel with 15 mL water. After extracting the water phase with 1-butanol (three times with 15 mL), the combined upper phases were diluted to 100 mL. Absorbance of this solution was measured at 545 nm with a Novaspec II (Pharmacia LKB). Each experiment was performed in triplicates. Content of procyanidins was calculated as cyanidin chloride with Equation (2) (A = absorbance at 545 nm; m = mass of examined substances (g)):

(2) $$\text{Content of procyanidins }\left(\text{\%}\right)=\frac{\text{A }\times \;200}{75\;\times \text{ m}}$$

Further information about the monomeric constitution of oligo- and polymeric procyanidins were generated via analytical HPLC and LC-ESI-HRMS.

Analytical HPLC: LaChrom Elite (Hitachi High-Technologies Europe GmbH, Krefeld, Germany) equipped with a L-2130 oven (adjusted to 35 °C), a L-2130 pump-cluster, a L-2200 autosampler and a L-2455 diode array detector with a LiChroCART^®^ 250-4, Purospher^®^ STAR RP-18e (5 µm) (Merck Chemicals GmbH), injection volume = 10 µL, absorbance = 525 nm, flow = 0.8 mL/min, mobile phase: 18% acetonitrile containing 0.02% TFA. Retention times of reference compounds: delphinidin 5.91 min, cyanidin 10.07 min and pelargonidin 18.28 min.

LC-ESI-HRMS: 1290 Infinity UHPLC (Agilent) equipped with a G4220A binary pump cluster, a G4226A auto sampler, a G1316C column oven (adjusted to 40.00 °C), a G4212A diode array detector (recorded range: 190–640 nm), Q-TOF 6540 UHD with dual ESI as ion source and acquisition range = 80–1400 *m*/*z* and a Zorbax Eclipse Plus C18, 1.8 µm, 50 × 2.1 mm column (Agilent). A = water with 0.1% formic acid, B = acetonitrile with 0.1% formic acid, flow = 0.6 mL/min, gradient: 0.0–4.0 min 5% → 98% B, 4.0–5.0 min 98% B isocratic, 5.0–5.1 min 98% → 5% B, 5.1–6.0 min 5% B isocratic.

### 3.10. Estimation of the Degree of Polymerization (mDP) via Thiolysis

Sample preparation: around 3.0 mg of a sample were subjected to a microwave-vial, dissolved in 300 µL ethanol, 30 µL benzyl mercaptan and 15 µL acetic acid. Each vial was beaded with an elastic septum and incubated for five days at 95 °C to achieve a complete degradation. Subsequently, the reaction mix was dried under nitrogen and dissolved in 1.00 mL ethanol. This preparation was filtered and analyzed by analytical HPLC: LaChrom Elite (Hitachi High-Technologies Europe GmbH) equipped with a L-2130 oven (adjusted to 25 C), a L-2130 pump-cluster, a L-2200 autosampler and a L-2455 diode array detector with a Hibar^®^ 250-4, Purospher^®^ STAR RP-18e (5 µm) column (Merck Chemicals GmbH); flow = 1 mL/min, absorbance = 280 nm. A = water with 0.02% trifluoroacetic acid, B = methanol/acetonitrile/water (50 + 40 + 10). Gradient: 0–10 min 5% B isocratic, 10–20 min 5% → 30% B, 20–48 min 30% → 70% B, 48–52 min 70% B isocratic, 52–54 min 70% → 100% B, 54–65 min 100% B isocratic, 65–67 min 100% → 5% B, 67–73 min 5% B isocratic.

To calculate the mDP, all peak areas of resulting reaction products were added (terminal and extending units) and divided through the areas belonging to the terminal units. Each reaction was performed in hexaplicates, calculation of the mDP was achieved by Equation (3) after thiolysis (mDP = mean degree of polymerization; AUC = area under the curve):

(3) $$\text{mDP}=\frac{\sum^{\text{​}}{\text{AUC}}_{\text{extending units}}\;+\sum^{\text{​}}{\text{AUC}}_{\text{terminal units}}}{\sum^{\text{​}}{\text{AUC}}_{\text{terminal units}}}$$

LC-ESI-HRMS: The equipment was the same like 3.9. with a LiChroCART^®^ 250-4, Purospher^®^ STAR RP-18e (5 µm) (Merck Chemicals GmbH) column, column oven (25 °C), Q-TOF 6540 UHD (Agilent) with dual ESI as ion source and an acquisition range from 80 to 1400 *m*/*z*, flow = 1 mL/min, A = water with 0.1% formic acid, B = methanol/acetonitrile/water (50 + 40 + 10); gradient: 0–10 min 5% B isocratic, 10–20 min 5% → 30% B, 20–48 min 30% → 70% B, 48–52 min 70% B isocratic, 52–54 min 70% → 100% B.

### 3.11. Phloroglucinol Degradation

The method was modified from Bicker *et al.* [32]. An aliquot of the sample was dissolved in a spillover of acidic ethanolic phloroglucinol solution (1.0 mL hydrochloric acid diluted with ethanol to 100.0 mL, 5.6 mg/mL phloroglucinol). This preparation was incubated without light for 30 min at room temperature. Finally the solution was dried under nitrogen or evaporation. The products of this cleavage were analyzed by LC-ESI-HRMS. The equipment used for the LC-ESI-HRMS was the same as described at 3.9., but the column was a YMC-Triart C18, 1.9 µm, 75 × 2 mm (YMC Europe GmbH, Dinslaken, Germany), column oven (25 °C), flow = 0.6 mL/min; A = water with 0.1% formic acid, B = acetonitrile with 0.1% formic acid; gradient: 0–12 min 0% → 40% B, 12–14 min 40% → 98% B, 14–14.1 min 98% → 0% B, 14.1–16 min 0% B isocratic.

### 3.12. Peracetylation

To verify and compare some structures by ^1^H-NMR with literature data, peracetylation was necessary. Samples were dissolved (0.5 mL pyridine and 0.5 mL acetic anhydride) and incubated without light and with moderate shaking for 24 h. Subsequently, the reaction product was precipitated by adding water. For further 12 h, the preparation was incubated in a fridge. To complete the procedure, the compound was washed three times with ice cooled water and finally dissolved in acetone. To dry the substance yielded by this extraction, the acetone was evaporated.

### 3.13. NMR Spectroscopy

All shift values (δ_H_ and δ_C_) are indicated in ppm, coupling constants in Hz. All free phenolic samples were measured in methanol-*d*_4_, the peracetylated compounds in chloroform-*d*_1_, and the proanthocyanidin enriched fractions in a mixture of methanol-*d*_4_/deuterium oxide (7 + 3). All spectra were referenced against residual undeuterated solvent. To get sharp NMR-signals from the samples showing rotary isomerism, low temperature measurements at 233 K and 253 K were necessary. These measurements were performed on a AVANCE III HD NMR (Bruker Corporation, Billerica, MA, USA) operating at 400.13 MHz (^1^H-NMR) and 100.63 MHz (^13^C-NMR). The spectra to the structures being accessible to NMR at room temperature were measured with a AVANCE III 600 NMR (Bruker Corporation) equipped with a Bruker 5 mm TCI CryoProbe operating at 600.25 MHz (^1^H-NMR) and 150.95 MHz (^13^C-NMR). For structure verification and elucidation, 1D-^1^H, 1D-^13^C, [^1^H-^13^C]-HSQC, [^1^H-^13^C]-HMBC, [^1^H-^1^H]-COSY and [^1^H-^1^H]-ROESY experiments were performed. The 1D-^1^H-NMR- and 1D-^13^C-NMR-spectra of the peracetates were performed on a AVANCE 300 (Bruker Corporation) operating at 300.13 MHz (^1^H-NMR) and 75.48 MHz (^13^C-NMR).

### 3.14. Mass Spectrometry

ESI-HRMS for structure elucidation and verification were performed on a Q-TOF 6540 UHD (Agilent).

### 3.15. Circular Dichroism

CD-spectra were acquired by a J-715 spectropolarimeter (JASCO Deutschland GmbH, Gross-Umstadt, Germany) operating at 22 °C. Area of measurement was 200–400 nm in 0.5 nm steps with 200 nm/min and 10 cycles. 1 mm Quartz cuvettes were used. Samples were all solved in methanol for spectroscopy, but the fraction containing the “unusual” PAs was solved in a mixture of methanol/water (80 + 20). Compounds **1** to **6** were concentrated at 175.0 µmol/L, compound **7** was concentrated at 36.6 µmol/L. The concentrations of the fractions S2 W and S3.8 were 0.06 mg/mL and 0.12 mg/mL respectively. The spectra were smoothed by the Savitzky-Golay algorithm with a convolution width of 15.

### 3.16. Polarimetry

Polarimetry of the isolated compounds was achieved by a UniPol L 1000 polarimeter (Schmidt + Haensch GmbH & Co., Berlin, Germany). The samples were solved in methanol for spectroscopy with a concentration of 0.1% and were measured at 589.30 nm in a micro tube (length = 50 mm, volume = 0.55 mL). To estimate the 2,3*-cis* to 2,3*-trans* ratio of the oligomeric and polymeric PAs, Equation (4) was used after determinating ${\left[\text{α}\right]}_{578}^{20}$ with a 341 Polarimeter (Perkin Elmer, Inc., Waltham, MA, USA) in a micro tube (length = 100 mm, volume = 1.0 mL; χ_cis_ = amount of 2,3*-cis* configurated flavan-3-ols included in a PA; ${\left[\text{α}\right]}_{578}^{20}$ = specific rotation of a PAs enriched fraction) [21,31]:

(4) $${\text{χ}}_{\text{cis}}\left(\%\right)=\frac{{\left[\text{α}\right]}_{578}^{20}+320}{480}\times 100$$

### 3.17. Isolated Compounds

*Epicatechin-(4β→8)-catechin, procyanidin B1* (**1**): 70.9 mg; ${\left[\text{α}\right]}_{589}^{23}$ +21.1; CD (methanol, Θ) 206 (–89460.7), 216 (+99040.8), 233.5 (+36458.0), 279.5 (−1236.81). Negative HR-ESI-MS *m*/*z* 577.1370 [M − H]^−^.

*Epicatechin-(4β→8)-epicatechin, procyanidin B2* (**2**): 11.7 mg; ${\left[\text{α}\right]}_{589}^{22}$ +55.6; CD (methanol, Θ) 205.5 (−88801.9), 216 (+74024.2), 236.5 (+30663.3), 273.5 (+2531.16). Negative HR-ESI-MS *m*/*z* 577.1358 [M − H]^−^.

*Catechin-(4α→8)-catechin, procyanidin B3* (**3**): 45.8 mg; ${\left[\text{α}\right]}_{589}^{23}$ −180.1; CD (methanol, Θ) 213.5 (−256750.0), 236 (−66990.6), 269.5 (+9984.63), 285 (+142.118). Negative HR-ESI-MS *m*/*z* 577.1369 [M − H]^−^.

*Catechin-(4α→8)-epicatechin, procyanidin B4* (**4**): 7.4 mg; ${\left[\text{α}\right]}_{589}^{23}$ −180.1; CD (methanol, Θ) 213 (−173964.0), 236 (−38178.2), 252 (+5365.08), 274 (+7867.0). Negative HR-ESI-MS *m*/*z* 577.1364 [M − H]^−^.

*Epicatechin-(4β→8)-epicatechin-(4β→8)-epicatechin, procyanidin C1* (**5**): 8.7 mg; ${\left[\text{α}\right]}_{589}^{23}$ +45.8; CD (methanol, Θ) 205.5 (−161056.0), 217 (+121294.0), 229.5 (+79868.8), 238 (+63979.8), 279 (−8569.24). Negative HR-ESI-MS *m*/*z* 865.2001 [M − H]^−^.

*Epicatechin-(4β→8)-epicatechin-(4β→8)-catechin* (**6**): 20.5 mg; ${\left[\text{α}\right]}_{589}^{23}$ +115.3; CD (methanol, Θ) 205.5 (−132845.0), 218 (+112144.0), 229.5 (+67413.4), 237 (+58945.7), 280 (−6730.77). Negative HR-ESI-MS *m*/*z* 865.1988 [M − H]^−^.

*Epicatechin-(4β→8)-epicatechin-(4β→8)-epicatechin-(4β→8)-catechin* (**7**): 15.3 mg; ${\left[\text{α}\right]}_{589}^{24}$ +74.9; CD (methanol, Θ) 206 (−237987.0), 219.5 (+220751.0), 237.5 (+102010.0), 248 (+25022.5), 279 (−11654.7). Negative HR-ESI-MS *m*/*z* 1153.2624 [M − H]^−^.

*Fraction S2 W* (“unusual” PAs): CD (methanol/water 80 + 20, Θ) 205.5 (−92820.8), 221.5 (+86787.4), 227 (+95101.3), 280.5 (−8054.69).

*Fraction S3.8* (“regular” PAs): ${\left[\text{α}\right]}_{578}^{20}$ +111.1; CD (methanol, Θ) 205.5 (−515604.0), 221.5 (+590460.0), 238 (+429040.0), 279.5 (−30794.0).

## 4. Conclusions

A systematic investigation of the proanthocyanidin pattern of *S. daphnoides* revealed the presence of four dimeric, two trimeric as well as a tetrameric procyanidin. In contrast to Pobłocka-Olech *et al.* [8], who purposed that willow bark does not contain procyanidin B2, this compound could be isolated. Furthermore two kinds of polar and presumably polymeric PA fractions (“regular” PAs and “unusual” PAs) were investigated and compared with different chromatographic and spectroscopic methods. A 2,3*-cis* configuration predominates in both fractions. The fraction with the “regular” PAs consists of slightly more tannins with more trihydroxylated constituent flavan-3-ol units and longer chains compared to the fraction with the “unusual” PAs. The identification of B-ring vicinal trihydroxylated flavan-3-ol units in polymeric PAs is in accordance with Foo and coworkers [33] who found them in *Salix caprea* L. However, that these observed slight differences of both fractions are responsible for the different chromatographic behavior on Sephadex^®^ LH-20 seems to be questionable. Further studies are necessary to satisfactorily describe chemical differences of the “unusual” and “regular” PA fractions.

## Supplementary Materials

Supplementary materials can be accessed at: http://www.mdpi.com/1420-3049/20/08/13674/s1.
