# Supplementary material for: Salix daphnoides: A Screening for Oligomeric and Polymeric Proanthocyanidins
Source: Molecules. 2015 Jul 29;20(8):13764–79. doi: 10.3390/molecules200813764 (PMC6332011; doi:10.3390/molecules200813764)
Supplement: Supplementary file 1 [file molecules-20-13764-s001.pdf]

Supplementary Materials

Table S1. NMR data for compounds **3** and **4**.

		**3** (PC B3)		**4** (PC B4)	
	Pos.	$\delta_{C,\ major}$ + $\delta_{C,\ minor}$ type	$\delta_{H,\ major}$ (J in Hz) + $\delta_{H,\ minor}$ (J in Hz)	$\delta_{C,\ major}$ + $\delta_{C,\ minor}$ type	$\delta_{H,\ major}$ (J in Hz) + $\delta_{H,\ minor}$ (J in Hz)
Upper unit	2	84.0 + 84.1, CH	4.25, d (9.7) + 4.35, d (9.1)	83.9 + 84.1, CH	4.41, d (9.7) + 4.31 [i], m
	3	73.7 [a] + 73.7 [a], CH	4.35, dd (9.7, 8.0) + 4.51, d (9.1)	73.8 + 73.8, CH	4,56, d (8.0) + 4.31 [i], m
	4	38.6 + 38.5, CH	4.40, d (7.9) + 4.49, d (8.0)	38.9 + 38.8, CH	4.62, d (7.9) + 4.46, d (7.1)
	4a	107.2 +107.1, C		107.2 + 107.4, C	
	5	157.1 [b], C		157.5 + 157.2, C	
	6	97.3 + 97.5, C	5.88, d (2.4) + 5.80, d (2.3)	97.6 + 97.7, CH	5.79, d (2.1) + 5.89, d (2.1)
	7	157.1 [b] + 157.3, C		157.3 + 157.3, C	
	8	96.9 + 96.2, CH	5.78, d (2.4) + 5.83, d (2.4)	96.1 + 97.1, CH	5.83, d (2.3) + 5.93, d (2.2)
	8a	158.6, C		158.7 + 158.6, C	
	1′	132.6 + 132.4, C		132.2 + 132.6, C	
	2′	116.4 + 116.2 [c], CH	6.73, d (1.9) + 6.95, d (1.9)	116.3 + 116.4, CH	6.98, d (1.9) + 6.69, d (1.9)
	3′	145.6 + 146.1 [d], C		146.5 + 145.6, C	
	4′	146.1 + 146.1, C		146.0 + 146.1, C	
	5′	116.2 + 115.9, CH	6.66, d (8.1) + 6.76, d (8.0)	116.0 [f] + 116.1 [f], CH	6.79 [j], d (7.7) + 6.60, d (8.2)
	6′	120.6 + 121.0, CH	6.46, dd (8.2, 1.9) + 6.81 [e], dd (8.0, 1.9)	121.2 + 120.5, CH	6.86, d (8.0) + 6.41, dd (8.2, 1.8)

Table S1. *Cont.*

		**3** (PC B3)		**4** (PC B4)	
	Pos.	$\delta_{C, major} + \delta_{C, minor}$ type	$\delta_{H, major}$ (J in Hz) + $\delta_{H, minor}$ (J in Hz)	$\delta_{C, major} + \delta_{C, minor}$ type	$\delta_{H, major}$ (J in Hz) + $\delta_{H, minor}$ (J in Hz)
Terminal unit	2	82.5 + 82.9, CH	4.53, d (7.3) + 4.74, d (7.3)	80.0 + 79.9, CH	4.93, bs + 4.80, bs
	3	68.9 + 68.6, CH	3.78, m + 4.06, m	67.4 + 67.8, CH	4.22, m + 4.05, m
	4	28.8 + 28.5, CH_2	2.48, dd (16.2, 8.0) + 2.58, dd (16.1, 7.7)<br>2.75, dd (16.3, 5.5) + 2.81, dd (16.2, 5.4)	30.1 + 29.4, CH_2	2.82, dd (16.9, 1.7) + 2.70, dd (17.1, 2.3)<br>2.92, dd (16.8, 4.4) + 2.86, dd (17.0, 5.0)
	4a	102.2 + 100.5, C		99.4 + 101.5, C	
	5	155.9 + 155.8, C		156.4 + 156.3, C	
	6	96.1 + 97.5, C	6.06, s + 5.93, s	97.5 + 96.4, CH	5.95, s + 6.09, s
	7	155.7, C		155.9 [g] + 155.9 [g], C	
	8	108.2 + 108.3, C		108.7 + 108.3, C	
	8a	154.9 + 155.0, C		155.4 [h] + 155.4 [h], C	
	1′	131.8 + 132.2, C		132.4 + 131.7, C	
	2′	115.5 + 115.2, CH	6.58, d (1.9) + 6.95, d (1.9)	115.3 + 114.8, CH	7,08, d (1.6) + 6.67, d (1.8)
	3′	145.5 + 146.1 [d], C		146.2 + 145.6, C	
	4′	145.8 + 146.1 [d], C		145.7 + 145.6, C	
	5′	116.1 + 116.1 [c], C	6.67, d (8.2) + 6.75, d (8.2)	116.1 + 115.9, CH	6.77 [j], d (8.2) + 6.71, d (8.2)
	6′	119.9 + 120.2, C	6.25, dd (8.2, 1.9) + 6.82 [e], dd (8.2, 1.8)	119.1 + 120.3, CH	6.86, d (8.0) + 6.44, dd (8.2, 1.8)

[a–j] Signals with the same letter are overlapped.

Table S2. NMR data for compounds **5** and **6**.

		**5** (PC C1)			**6** (Epicatechin-β(4→8)-epicatechin-β(4→8)-catechin)		
	Pos.	δ_C type (−40 °C)	δ_H (*J* in Hz) (−40 °C)	δ_H (*J* in Hz) (−20 °C)	δ_C type (−40 °C)	δ_H (*J* in Hz) (−40 °C)	δ_H (*J* in Hz) (−20 °C)
upper unit	2	76.8, CH	5.08, bs	5.07, bs	76.7, CH	5.08 [f], bs	5.07, bs
	3	73.5, CH	3.95, d (1.9)	3.97, d (1.9)	73.5, CH	3.98, d (1.6)	3.99, d (1.8)
	4	36.9, CH	4.68, bs	4.69, m	36.8, CH	4.67, bs	4.68, bs
	4a	101.4, C			101.9, C		
	5	157.2 [l], C			158.0 [p], C		
	6	95.7 [m], CH	5.96 [n], d (2.3)	5.97 [o], d (2.4)	95.8 [q], CH	5.95 [r], d (2.3)	5.97 [s], d (2.4)
	7	158.3, C			157.8 [e], C		
	8	95.8 [m], CH	5.98 [n], d (2.3)	6.00 [o], d (2.3)	95.8 [q], CH	5.99 [r], d (2.3)	5.98 [s], d (2.4)
	8a	157.4 [l], C			157.8 [e,m], C		
	1′	132.6, C			132.5, C		
	2′	114.9, CH	6.89 [a], d (1.7)	6.89 [c], d (1.8)	114.8, CH	6.90 [g], d (1.6)	6.90 [i], d (1.8)
	3′	145.6, C			145.6, C		
	4′	145.3, C			145.3, C		
	5′	115.7, CH	6.73, d (8.2)	6.73 [d], d (8.1)	115.7, CH	6.73 [h], d (8.3)	6.74 [j], d (8.0)
	6′	118.9, CH	6.64–6.70 [b]	6.70 [d]	118.9, CH	6.66–6.70 [h]	6.67–6.72 [j]
Middle unit	2	76.7, CH	5.26, bs	H_2O-signal	76.8, CH	5.31, bs	5.29, bs
	3	73.2, CH	3.90, d (2.1)	3.93, d (2.0)	72.4, CH	4.07, d (1.6)	4.07, d (1.7)
	4	37.1, CH	4.68, bs	4,69, m	37.0, CH	4.77, bs	4.76, bs
	4a	102.0, C			102.5, C		
	5	157.0, C			157.0, C		
	6	96.6, CH	5.88, s	5.89, s	96.6, CH	5.87, s	5.88, s
	7	155.9, C			156.8, C		
	8	106.8, C			106.2, C		
	8a	154.1, C			154.9, C		
	1′	132.6, C			132.8, C		
	2′	114.8, CH	7.02, d (1.3)	7.02, d (1.6)	114.6, CH	7.04, d (1.5)	7.04, d (1.7)
	3′	145.8,C			145.7, C		
	4′	145.3, C			145.1, C		
	5′	115.7, CH	6.64–6.70 [b]	6.71 [d], d (8.6)	115.7, CH	6.68 [h], d (8.3)	6.69, d (8.2)
	6′	118.4, CH	6.64–6.70 [b]	6.69 [d], d (6.9)	118.5, CH	6.78, dd (8.1, 1.7)	6.79, dd (8.2, 1.9)

Table S2. *Cont.*

		5 (PC C1)			6 (Epicatechin-β(4→8)-epicatechin-β(4→8)-catechin)		
	Pos.	δ_C type (−40 °C)	δ_H (*J* in Hz) (−40 °C)	δ_H (*J* in Hz) (−20 °C)	δ_C type (−40 °C)	δ_H (*J* in Hz) (−40 °C)	δ_H (*J* in Hz) (−20 °C)
Terminal unit	2	79.4, CH	4.99, bs	4.99, bs	81.3, CH	5.06 [f], d (4.6)	5.02, d (4.7)
	3	66.8, CH	4.30, m	4.31, m	68.0, CH	4.21, q (4.7)	4.19, dd (10.2, 5.1)
	4	29.9, CH_2	2.80, d (16.1)<br>2.95, dd (16.6, 4.1)	2.81, dd (16.9, 1.8)<br>2.95, dd (16.6, 4.1)	25.9, CH_2	2.50, dd (15.7, 2.8)<br>2.62, dd (16.4, 4.4)	2.56 [k], dd (16.6, 4.9)<br>2.62 [k], dd (16.9, 5.2)
	4a	99.7, C			99.8		
	5	156.9, C			155.7, C		
	6	97.0, CH	5.93, s	5.93, s	96.8, CH	5.89, s	5.89, s
	7	156.1, C			156.4, C		
	8	107.5, C			108.1, C		
	8a	154.1, C			153.6, C		
	1′	132.0, C			132.3, C		
	2′	115.0, CH	7.10, d (1.7)	7.11, d (1.8)	113.5, CH	6.83, d (1.4)	6.86, d (1.8)
	3′	146.0, C			146.0, C		
	4′	145.3, C			145.6, C		
	5′	115.6, CH	6.74, d (8.2)	6.75, d (8.2)	115.9, CH	6.71 [h], d (8.3)	6.72 [j], d (8.0)
	6′	118.7, CH	6.88 [a], dd (8.0, 1.5)	6.90 [c], dd (8.2, 1.9)	119.0, CH	6.92 [g], dd (8.5, 1.6)	6.90 [i], dd (8.1, 1.8)

[a–k] Signals with the same letter are overlapped; [l–s] Signals with the same letter are interchangeable.

Table S3. NMR data for compound **7**.

	Pos.	**7** (Epicatechin-*β*(4→8)-epicatechin-*β*(4→8)-epicatechin-*β*(4→8)-catechin)		
		δ_C type (−40 °C)	δ_H (*J* in Hz) (−40 °C)	δ_H (*J* in Hz) (−20 °C)
Upper unit	2	76.5, CH	5.28, bs	H_2O-signal
	3	73.2, CH	4.08, d (1.6)	4.10 [g], d (2.1)
	4	37.3, CH	4.74, bs	4.75, bs
	4a	102.3, C		
	5	157.9 [a,k], C		
	6	95.7 [l], CH	5.96 [m], d (2.1)	5.98 [n], d (2.3)
	7	158.9, C		
	8	95.8 [l], CH	5.99 [m], d (2.2)	6.01 [n], d (2.3)
	8a	157.9 [a,k], C		
	1′	132.6, C		
	2′	114.7, CH	7.11, d (1.0)	7.10, d (1.6)
	3′	145.7 [b], C		
	4′	145.1, C		
	5′	115.7, CH	6.69–6.72 [d]	6.70 [h], d (8.1)
	6′	118.4, CH	6.75 [d], m*	6.77, dd (8.5, 1.7)
Upper middle unit	2	76.7, CH	5.09 [e], bs	5.08, bs
	3	73.6, CH	3.97, d (1.6)	3.99, d (1.8)
	4	36.9, CH	4.72, bs	4.72, bs
	4a	102.1, C		
	5	157.3, C		
	6	96.8, CH	5.92, s	5.93, s
	7	156.7, C		
	8	106.5, C		
	8a	155.0, C		
	1′	132.5, C		
	2′	114.9, CH	6.90, d (1.6)	6.90 [i], d (1.5)
	3′	145.8 [c], C		
	4′	145.3, C		
	5′	115.7, CH	6.74 [d], m	6.74 [h], d (8.1)
	6′	118.9, CH	6.68 [d], dd (8.1, 1.3)	6.69 [h], dd (8.5, 1.7)

Table S3. *Cont.*

	Pos.	**7** (Epicatechin-*β*(4→8)-epicatechin-*β*(4→8)-epicatechin-*β*(4→8)-catechin)		
		δ_C type (−40 °C)	δ_H (*J* in Hz) (−40 °C)	δ_H (*J* in Hz) (−20 °C)
Lower middle unit	2	76.8, CH	5.37, bs	5.34, bs
	3	72.1, CH	4.12, d (1.3)	4.11 [g], d (2.1)
	4	37.1, C	4.79, bs	4.78, bs
	4a	102.7, C		
	5	157.7, C		
	6	96.8, CH	5.93, s	5.92, s
	7	156.4, C		
	8	107.0, C		
	8a	154.8, C		
	1′	132.7, C		
	2′	114.8, CH	7.04, d (1.4)	7.05, d (1.7)
	3′	145.8 [c], C		
	4′	145.2, C		
	5′	115.7, CH	6.70 [d], m	6.72 [h], m
	6′	118.6, CH	6.82 [f], m	6.82, dd (8.3, 1.2)
Terminal unit	2	81.3, CH	5.08 [e], d (4.5)	5.04, d (5.0)
	3	68.0, CH	4.22, m	4.19, m
	4	25.9, CH_2	2.50, dd (16.5, 4.6)	2.56 [j], dd (16.5, 4.6)
			2.62, dd (16.7, 4.6)	2.63 [j], dd (16.7, 4.6)
	4a	99.8, C		
	5	155.7, C		
	6	96.5, CH	5.89, s	5.89, s
	7	157.9 [a], C		
	8	108.1, C		
	8a	153.6, C		
	1′	132.3, C		
	2′	113.5, CH	6.84 [f], d (1.3)	6.87, d (1.8)
	3′	146.1, C		
	4′	145.7 [b], C		
	5′	115.7, CH	6.71[d], m,	6.72 [h], m
	6′	119.0, CH	6.93, dd (8.5, 1.3)	6.91 [i], dd (8.4, 1.7)

[a–j] Signals with the same letter are overlapped; [k–n] Signals with the same letter are interchangeable.

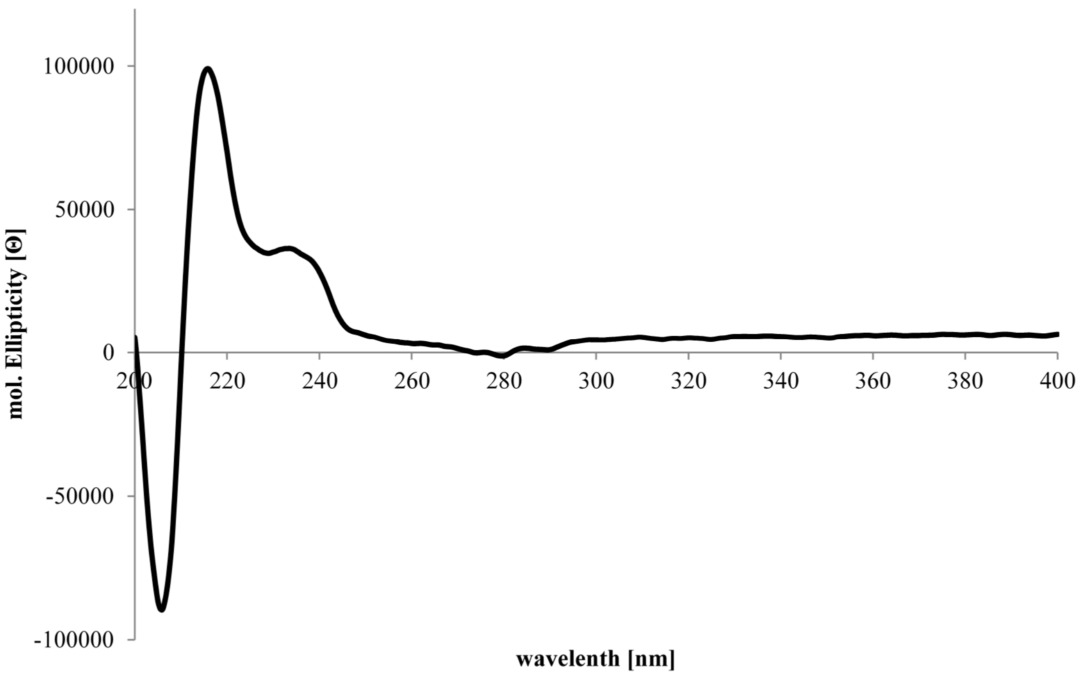

Figure S1. CD-spectrum of compound **1**.

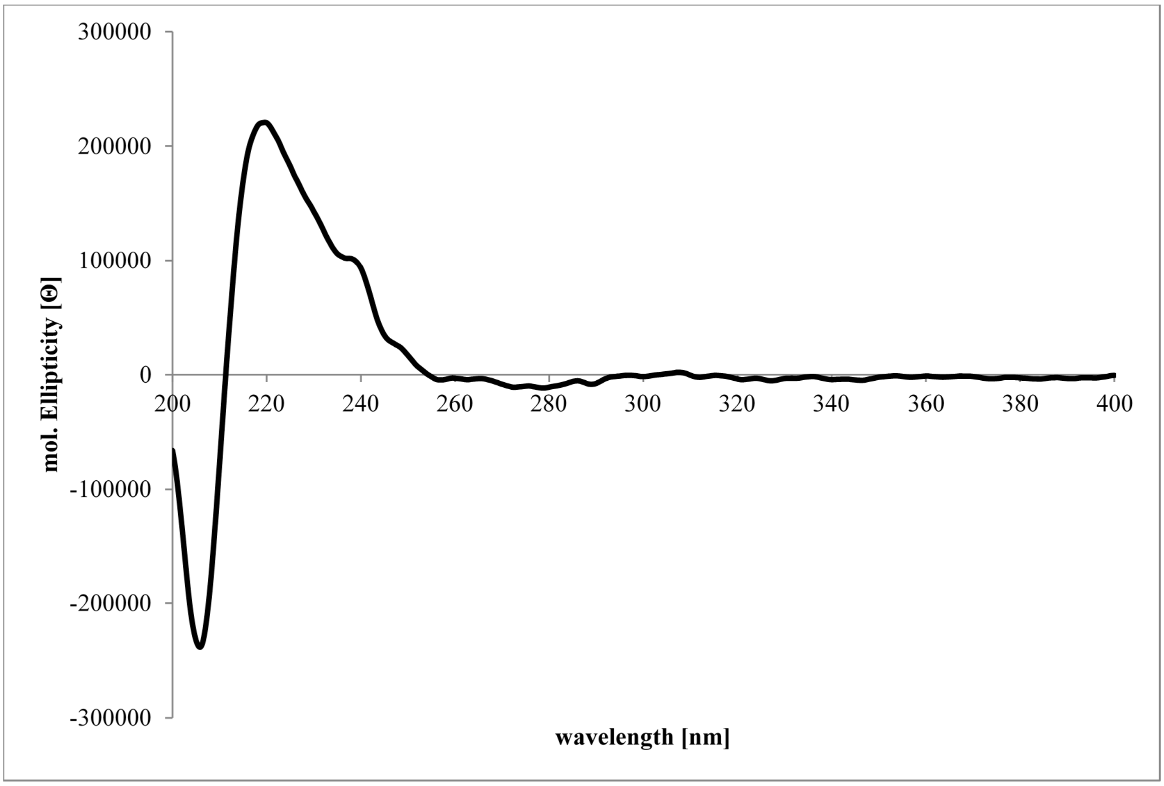

Figure S2. CD-spectrum of compound **7**.

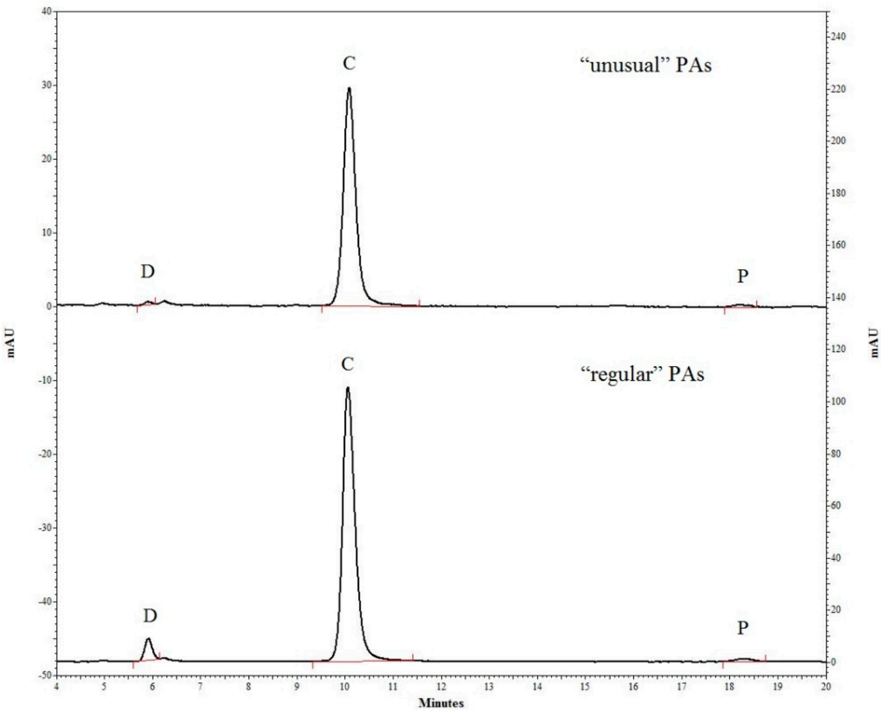

Figure S3. HPLC chromatograms after the cleavage reaction achieved by HCl/O_2. The left y-axis indicates the absorbance of the upper chromatogram (“unusual” PAs) and the right y-axis for the lower one (“regular” PAs) respectively. D = delphinidin, C = cyanidin, P = pelargonidin.

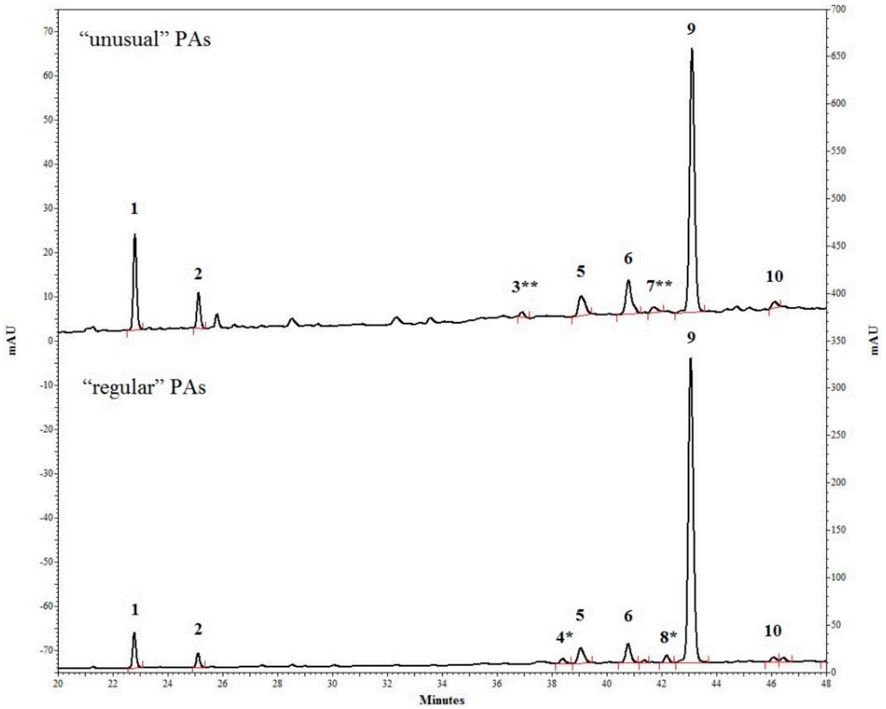

Figure S4. HPLC chromatograms after thiolysis. The left y-axis indicates the absorbance of the upper chromatogram (“unusual” PAs) and the right y-axis for the lower one (“regular” PAs), respectively. The peak numbers are explained in Table 2 of the main text.
